# A Phase I, Randomized, Single-Dose Study Evaluating the Biosimilarity of TAB008 to Bevacizumab in Healthy Volunteers

**DOI:** 10.3389/fphar.2019.00905

**Published:** 2019-08-15

**Authors:** Jin Wang, Lu Qi, Long Liu, Zejuan Wang, Gang Chen, Yu Wang, Xiaona Liu, Ying Liu, Huijuan Liu, Yuanxu Tong, Chen Liu, Chunpu Lei, Xinghe Wang

**Affiliations:** Phase I Clinical Trial Center, Beijing Shijitan Hospital, Capital Medical University, Beijing, China

**Keywords:** TAB008 monoclonal antibody injection, Avastin®, pharmacokinetics, biosimilarity, immunogenicity

## Abstract

**Objective:** This study compared the pharmacokinetics (PK), safety, and immunogenicity of the biosimilar TAB008 monoclonal antibody to bevacizumab (Avastin^®^) in normal healthy Chinese male volunteers.

**Methods:** In this randomized, double-blind, parallel controlled study, a total of 100 healthy Chinese male subjects were randomized (1:1) to receive a single 1 mg/kg intravenous dose of TAB008 or Avastin^®^ over a 90-min infusion. The subjects were followed for 99 days after drug administration. Primary endpoints were bioequivalence of major pharmacokinetic parameters (AUC_0-t_ and AUC_0-∞_) and maximum observed serum concentration (C_max_). Secondary endpoints included safety and immunogenicity parameters.

**Results:** The two groups of test subjects (49 subjects in the TAB008 group and 50 subjects in the Avastin^®^ group) were well matched in regards to all demographic and baseline characteristics. The treatment group ratios of LS geometric means for the three primary PK parameters were fully contained within the bioequivalence limits of 80.00–125.00% (90% CI was 103.66–118.33% for C_max_, 94.32–111.72% for AUC_0-t_, and 94.69–112.23% for AUC_0-∞_). Treatment-emergent adverse events (TEAEs) were reported for 24 (49.0%) subjects in the TAB008 group and 22 (44.0%) subjects in the Avastin^®^ group. TEAEs related to the study drug were reported for 19 (38.8%) subjects in the TAB008 group and 19 (38.0%) subjects in the Avastin^®^ group. National Cancer Institute-Common Terminology Criteria for Adverse Events (NCI-CTCAE) Grade 3 TEAEs were reported for 1 (2.0%) subject in the TAB008 group and 3 (6.0%) subjects in the Avastin^®^ group. There were no Grade 4 or 5 TEAEs or serious adverse events (SAEs) during the study. Anti-drug antibody generation was reported once only in each group, and neutralizing antibody (Nab) analysis was negative upon follow-up.

**Conclusion:** TAB008 attained pharmacokinetic similarity to bevacizumab, and was safe and well tolerated.

## Introduction

Bevacizumab (Avastin^®^) is a recombinant humanized monoclonal IgG_1_ antibody that effectively binds to and inhibits vascular endothelial growth factor (VEGF), thereby reducing new blood vessel formation (Avastin: EPAR-Product Information. EMA; US FDA Avastin®). Avastin^®^ was first approved by the United States Food and Drug Administration (FDA) in February 2004 for first-line treatment of metastatic colorectal cancer in combination with chemotherapy, and then for first-line treatment of advanced non-squamous non-small cell, non-EGFR-mutant lung cancer (NSCLC) in combination with paclitaxel and carboplatin. In 2009, Avastin was approved for glioblastoma and renal cell carcinoma after prior therapy. In 2014, significant efficacy was demonstrated against cervical and ovarian cancer. All these approvals were based on stringent randomized clinical trials with significant overall survival improvement as the cornerstone for approval. In the European Union, aside from the previously mentioned indications, the EMA also approved Avastin^®^ in combination with EGFR tyrosine kinase inhibitors as first-line therapy in EGFR-mutant NSCLCs, and first-line treatment in combination with paclitaxel or capecitabine (if paclitaxel-intolerant) for triple-negative breast cancer. Avastin^®^ has since been approved in many countries and regions in the world (Avastin: EPAR-Product Information. EMA; US FDA Avastin®).

In China, a phase I dose escalation study (5, 10, 15 mg/kg) was performed in 39 refractory solid tumor patients. Bevacizumab pharmacokinetics were dose-linear at 5 mg/kg every 2 weeks and 15 mg/kg every 3 weeks (Wu et al., 2010). In February 2010, Avastin^®^ (5 mg/kg every 2 weeks) was approved in combination with irinotecan, 5-fluorouracil, and leucovorin for first-line management of metastatic colorectal cancer after the registration ARTIST trial (214 patients) demonstrated improved efficacy compared with patients who received chemotherapy alone; outcomes included overall response rate (ORR) (35% *vs.* 17%, respectively; p = 0.013), progression-free survival (PFS) (8.3 *vs.* 4.2 months, respectively; p < 0.01), and overall survival (OS) (18.7 *vs.* 13.4 months, respectively; p = 0.014) (Guan et al., 2011). In July 2015, the China Food and Drug Administration approved bevacizumab (15 mg/kg every 3 weeks) in combination with paclitaxel and carboplatin for first-line management of NSCLC after the registration BEYOND study (276 patients) demonstrated improved ORR (54% *vs.* 26%, p < 0.01), PFS (9.2 *vs.* 6.5 months, p < 0.01), and OS (24.3 *vs.* 17.7 months, HR = 0.68, 95% CI 0.5–0.93, p = 0.0154) compared with patients who received chemotherapy alone (Zhou et al., 2015).

Bevacizumab has demonstrated powerful anticancer efficacy when added to chemotherapy. Recent research suggests that in combination with checkpoint inhibitors, Avastin further expands the landscape of cancer control (Socinski et al., 2018). As a result of bevacizumab’s great potential, many biosimilars have been developed globally.

The abbreviated licensure pathway for development of biosimilars has been clearly outlined by regulatory authorities, including the EMA (issued October 23, 2014) (Avastin: EPAR-Product Information. EMA), the US FDA (issued April 2015) (US FDA Avastin®), and China (Technical Guidelines for Development and Evaluation of Biosimilars, issued February 28, 2015 by the Center for Drug Evaluation) (Technical guidelines for research and evaluation of biosimilars. NMPA). China also issued an additional directive on July 18, 2017, specifically for bevacizumab (Recommendations on the design and review of clinical study for bevacizumab injection biosimilar drug). All guidelines require a phase I comparative pharmacokinetic study to the originator, with concurrent evaluation of the safety and immunogenicity profile of the biosimilar product. Phase I clinical trials should be followed by phase III clinical trials with overall response rate as primary endpoint.

TAB008 is a biosimilar monoclonal antibody to bevacizumab developed by TOT BIOPHARM. Analytical similarities have been demonstrated in comparative physical, chemical, and extensive structural and functional characterization studies of TAB008 and the originator bevacizumab. Pre-clinical, pharmacokinetic, pharmacologic, and toxicologic studies (in mice, rats, and cynomolgus monkeys), with Avastin^®^ as the control, confirmed TAB008 to be a biosimilar.

Pharmacokinetic studies demonstrated that bevacizumab showed dose linearity within the dose range of 1–20 mg/kg, with a terminal t_½_ of around 20 days (Han et al., 2016). As such, a single 1 mg/kg dose over a 90-min infusion was selected for administration in a phase I randomized, double-blind, bevacizumab (EU-sourced) controlled study to compare the pharmacokinetics, safety, and immunogenicity of TAB008 versus Avastin^®^ in healthy Chinese male subjects. The 1 mg/kg dose was selected to minimize drug exposure in normal healthy volunteers, so such a low dose over a 90-min infusion will be a very stringent test for the biosimilarity of TAB008. Since the main purpose of any biosimilar study is to measure similarities, the FDA, EMA, and National Medical Products Administration (NMPA) suggest choosing healthy homogeneous male population to describe PK characteristics (Avastin: EPAR-Product Information. EMA; Recommendations on the design and review of clinical study for bevacizumab injection biosimilar drug; US FDA Avastin®).

## Materials, Subjects, and Methods

### Study Population

The study was registered in the Chinese Clinical Trials Registry (ChiCTR-IIR-16009827). The trial protocol was approved by the Drug Clinical Trial Ethics Committee of Beijing Shijitan Hospital of Capital Medical University. All subjects provided written informed consent. A total of 100 healthy Chinese male subjects were randomized (1:1) to receive a single 1 mg/kg intravenous dose of either TAB008 or Avastin^®^. Inclusion criteria were as follows: 18–45 years of age, body mass index (BMI) of 19–28 kg/m^2^ (inclusive), and body weight of 50–75 kg (inclusive). The subjects were evaluated through medical history, physical examination, laboratory tests, and electrocardiogram (ECG). Exclusion criteria included history of significant illness, alcohol or drug abuse (all volunteers underwent a drug abuse screen), prior exposure to bevacizumab or any VEGF- or VEGF receptor-targeting drug, or participation in clinical trials within the past 3 months. Patients with detectable baseline anti-bevacizumab antibody were also excluded.

### Study Design

The study was a phase I, single-dose, randomized, double-blind, parallel controlled study designed to investigate the pharmacokinetics, safety and immunogenicity of TAB008 Monoclonal Antibody Injection compared to Avastin^®^ in healthy Chinese male subjects.

### Pharmacokinetics Analysis

Pharmacokinetic parameters were calculated using non-compartmental methods (Phoenix^®^ WinNonlin^®^ 6.4) and summarized using descriptive statistics. The primary PK parameters C_max_, AUC_0-t_, and AUC_0-∞_ were compared between treatment groups using a linear model. Secondary endpoints evaluated included t_max_ (time to maximum serum concentration), CL (systemic clearance), z (terminal rate constant), t½ (terminal half-life), Vss (volume of distribution at steady state), and Vz (volume of distribution).

Blood samples were collected from each subject at pre-determined time points (pre-dosing; 0.75 h after initiation of infusion; 0, 1, 2, 4, and 8 h from the end of infusion; and at 24, 48, and 96 h; and days 8, 11, 15, 22, 29, 36, 43, 57, 71, 85, and 99 from the start of infusion). Blood (4 ml) was drawn at each time point; 21 blood samples were collected (84 ml in total). Serum drug concentration was measured using a validated enzyme-linked immunosorbent assay. The quantification range of the assay was 39.06–10,000.00 ng/ml, with lower limit of quantification of 78.13 ng/ml.

### Safety Evaluation

Safety parameters included reporting of adverse events (AE) and abnormal clinical laboratory test values, ECG, vital signs, physical examinations, and immunogenicity tests. The number of subjects with AEs was summarized, coded, and classified according to the Medical Dictionary for Regulatory Activities (MedDRA) (version 20.0). AEs were graded according to CTCAE (version 4.03). Descriptive statistical analyses were applied.

### Immunogenicity

TAB008 Monoclonal Antibody Injection and Avastin^®^ were compared in terms of immunogenicity. Anti-drug antibody (ADA) was screened for as scheduled (pre-dosing and days 15, 43, and 85 from the start of infusion). Samples were first tested for binding to TAB008 Monoclonal Antibody Injection to identify potential presence of ADAs; if the result was positive, a confirmatory test based on TAB008 Monoclonal Antibody Injection and the reference Avastin^®^ was conducted to confirm the positive status. A third test was performed on the confirmed ADA-positive samples to test the relative titer of ADA. If the sample tested was confirmed as ADA-positive, Nab analysis was conducted.

### Sample Size Estimation

When calculating the main endpoints of AUC_0-∞_, AUC_0-t_, and C_max_, an inter-individual difference of 25% was presumed so that the ratio of the true mean would be 1.05. Considering 80.00–125.00% equivalence margin for the main PK parameters and α = 0.05 (double unilateral, t-test), 36 effective subjects were required in each treatment group. Considering prior clinical trial data and a 10% dropout rate, 45 subjects were needed per arm. After 90 subjects were treated under blinded conditions, the sample size was re-estimated. If the coefficient of variation (cv) was ≤28%, recruitment continued up to a total of no more than 100 subjects, but if the cv was >28%, and the recalculated sample size was ≤285, the study continued to recruit up to the recalculated sample number. Sample size estimation was performed using SAS 9.2.

### Statistical Considerations

Pharmacokinetic similarity between two drugs can be declared if the 90% CIs of the ratios for the geometric least-squares (LS) means of the primary PK parameters (AUC_0-∞_, AUC_0-t_, and C_max_) are within the range 80.00–125.00%.

## Results

### Disposition and General Characteristics of the Study Subjects

**Figure 1:**
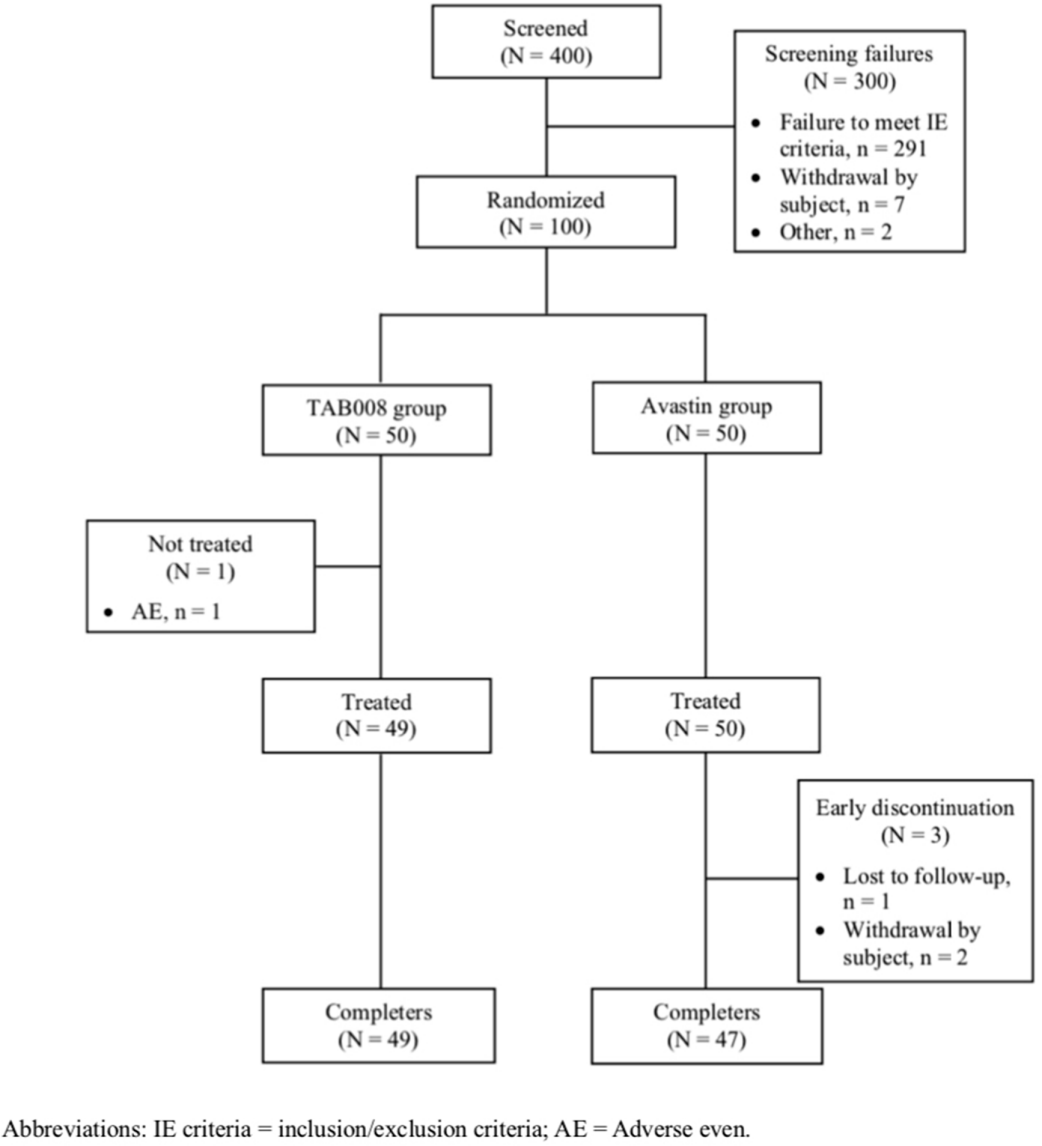
Subjects dispositions for TAB008 group versus Avastin^®^ group.

A total of 400 subjects signed the informed consent form. There were 300 screen failures (291 failed to fulfill enrollment criteria and 7 withdrew after providing consent). Of the 100 subjects randomized, 49 received TAB008 (one subject experienced a non-drug related AE) and 50 received Avastin^®^ and were included in the full analysis set (FAS) (**Figure 1**). All subjects in the FAS were also included in the pharmacokinetics analysis set (PKAS) and safety analysis set (SAF). Eventually, 49 subjects in the TAB008 group and 47 subjects in the Avastin^®^ group completed the study. There were three early discontinuations in the Avastin^®^ group: withdrawal of consent (Avastin: EPAR-product Information. EMA) and loss to follow-up (US FDA Avastin®).

The two groups were well matched with regard to all demographic and baseline characteristics. Mean age was 29.5 (± 5.42) years in the TAB008 group and 29.3 (± 5.59) years in the Avastin^®^ group. Mean BMI was 22.40 (± 1.90) kg/m^2^ in the TAB008 group and 22.14 (± 1.89) kg/m^2^ in the Avastin^®^ group. The two groups had a comparable history of tobacco and alcohol use. No abnormalities were with regard to baseline vital signs or ECG or physical examination results.

### Pharmacokinetic Parameters

**Figure 2:**
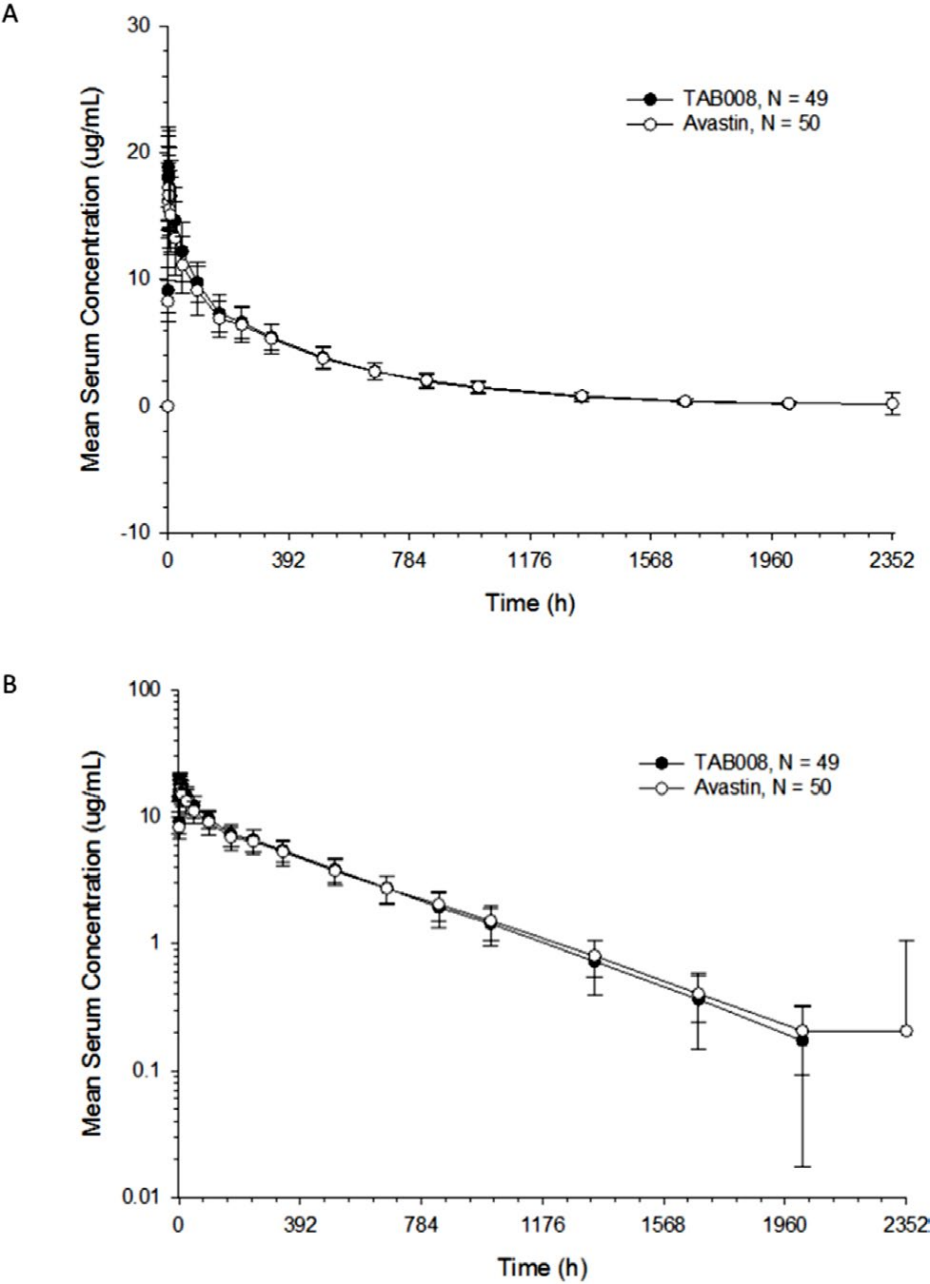
Arithmetic mean (± SD) bevacizumab serum concentration–time profiles for TAB008 versus Avastin^®^ on linear **(A)** and semilogarithmic scales **(B)**.

All PK analyses were carried out using the PKAS, which comprised subjects who received a full dose of study drug and had at least one valid post-dose concentration detected without significant protocol deviations or events that could seriously impact PK concentration. PK parameters are shown in **Figure 2** and **Table 1**. The data show that the primary and secondary PK parameters were similar between the two treatment groups.

**Table 1:** Descriptive statistics for geometric mean and geometric CV of pharmacokinetic parameters for each treatment group.

Parameter (unit)	TAB008 (N = 49)		Avastin^®^ (N = 50)	
	n	Geometric mean (CV%)^a^	n	Geometric mean (CV%)^a^
AUC_0-∞_ (炜µg·h/ml)	49	5,523 (20.8)	46	5,358 (29.4)
AUC_0-t_ (炜µg·h/ml)	49	5,447 (20.6)	47	5,306 (29.5)
C_max_ (炜µg/ml)	49	19.25 (16.5)	50	17.38 (23.1)
t_max_ (h)	49	2.50 (1.50, 5.50)	50	2.50 (1.50, 9.50)
t_1/2_ (h)	49	321.4 (21.4)	46	345.1 (15.3)
CL (L/h)	49	0.01160 (21.6)	46	0.01182 (29.4)
V_z_ (L)	49	5.384 (17.1)	46	5.885 (28.0)
V_ss_ (L)	49	5.342 (16.1)	46	5.857 (25.2)

CV, coefficient of variation.

a t_max_ is presented as median (minimum, maximum).

### Bioequivalence Statistical Results

The mean concentration profiles between TAB008 Monoclonal Antibody Injection and Avastin^®^ were similar over the profiling interval. The treatment group ratios of LS geometric means for the three primary PK parameters were fully contained within the bioequivalence limits of 80.00–125.00% (90% CI was 103.66–118.33% for C_max_, 94.32–111.72% for AUC_0-t_, and 94.69–112.23% for AUC_0-∞_) (**Table 2**). Based on the result of the nonparametric analysis, the median t_max_ values of TAB008 Monoclonal Antibody Injection and Avastin^®^ were the same.

**Table 2:** Statistical comparison of primary pharmacokinetic parameters.

Parameter (unit)	Treatment	n	Geometric LS Means	95% CI	Pairwise comparison		
					Pair	Ratio (%)	90% CI
AUC_0-∞_ (h·µg/ml)	Avastin^®^	46	5,358	(4,981, 5,763)	TAB008/ Avastin^®^	103.09	(94.69, 112.23)
	TAB008	49	5,523	(5,146, 5,927)			
AUC_0-t_ (h·µg/ml)	Avastin	47	5,306	(4,936, 5,704)	TAB008/ Avastin^®^	102.65	(94.32, 111.72)
	TAB008	49	5,447	(5,075, 5,847)			
C_max_ (µg/ml)	Avastin	50	17.38	(16.44, 18.38)	TAB008/ Avastin^®^	110.77	(103.66, 118.33)
	TAB008	49	19.25	(18.20, 20.37)			

CI, confidence interval; LS, least-squares.

### Safety Analysis Results

TAB008 Monoclonal Antibody Injection was safe and well tolerated in healthy Chinese male subjects and demonstrated safety profiles comparable with those of Avastin^®^. TEAEs were reported for 24 (49.0%) subjects in the TAB008 group and 22 (44.0%) subjects in the Avastin^®^ group. TEAEs considered by the investigator to be related to the study drug were reported for 19 (38.8%) subjects in the TAB008 group and 19 (38.0%) subjects in the Avastin^®^ group. NCI-CTCAE Grade 3 TEAEs were reported for one (2.0%) subject in the TAB008 group and three (6.0%) subjects in the Avastin^®^ group. There were no Grade 4 or 5 TEAEs, SAEs, or deaths during the study, and no TEAEs leading to treatment discontinuation (**Table 3**).

**Table 3:** Summary of all treatment-emergent adverse events; grade 3 AE detailed.

Category	TAB008 (N = 49) n (%)	Avastin^®^ (N = 50) n (%)
Subjects with		
At least 1 TEAE	24 (49.0)	22 (44.0)
At least 1 TEAE related to study drug	19 (38.8)	19 (38.0)
At least 1 Grade 3 or above TEAE	1 (2.0)	3 (6.0)
At least 1 Grade 3 or above TEAE related to study drug	1 (2.0)	3 (6.0)
Amylase increased	1 (2.0)	0 (0.0)
Blood triglycerides increased	0 (0.0)	1 (2.0)
Blood creatine phosphokinase increased	0 (0.0)	1 (2.0)
Hyperuricemia	0 (0.0)	1 (2.0)
At least 1 SAE	0 (0.0)	0 (0.0)
Deaths due to TEAE	0 (0.0)	0 (0.0)

TEAE, treatment-emergent adverse event; SAE, serious adverse event.

Subjects with more than 1 event of the same TEAE are counted once at the worst severity or strongest relationship category.

Hypertriglyceridemia was the most frequently reported treatment-related AE in both the TAB008 (10.2%) and Avastin^®^ (8.0%) groups. Clinically significant increases in triglycerides, uric acid, and liver function test results (alanine aminotransferase, total bilirubin, and direct bilirubin) were observed in both groups. Clinically significant hypertension was observed for both groups (**Table 4**). ECG and physical examination did not reveal any clinically relevant abnormalities in either group.

**Table 4:** Summary of most frequent (>2%) treatment-related treatment-emergent adverse events by system organ class and preferred term for each treatment group (safety analysis set).

System organ class Preferred term	TAB008 (N = 49) n (%)	Avastin (N = 50) n (%)
Subjects with at least 1 treatment-related TEAE	19 (38.8)	19 (38.0)
Blood triglycerides increased	5 (10.2)	4 (8.0)
Bilirubin conjugated increased	3 (6.1)	2 (4.0)
Blood bilirubin unconjugated increased	3 (6.1)	2 (4.0)
Alanine aminotransferase increased	2 (4.1)	2 (4.0)
Blood bilirubin increased	2 (4.1)	2 (4.0)
Blood pressure diastolic increased	2 (4.1)	0 (0.0)
Blood creatine phosphokinase increased	0 (0.0)	2 (4.0)
Epistaxis	3 (6.1)	1 (2.0)
Upper respiratory tract infection	2 (4.1)	1 (2.0)

TEAE, treatment-emergent adverse event; ALT, alanine aminotransferase.

Subjects with more than 1 event of the same TEAE are counted once at the worst severity or strongest relationship category.

### Immunogenicity Examination Findings

All subjects tested negative for ADA before treatment. One subject in the TAB008 group and one in the Avastin^®^ group showed positive ADA on days 15 and 85, respectively, but positivity disappeared at the subsequent testing time point. The ADAs were not neutralizing antibodies. Neutralizing antibody titers were not assessed for other subjects.

## Discussion

According to the FDA, EMA, and NMPA guidelines, all biosimilars of bevacizumab used in phase I trials have to be compared to the originator, which can be sourced from Europe and/or the US. Avastin^®^ (EU-sourced) was approved by NMPA in 2010 for the treatment of colorectal cancer, NSCLC, malignant glioma, and other tumors in China; therefore, Avastin^®^ (EU-sourced) was chosen as the control for this study.

Phase I studies for evaluating biosimilarity to originator molecules have been conducted in some countries. The doses ranged from 1–5 mg/kg with infusion times of 30–90 min, and the number of normal healthy male volunteers ranged from 24–68 per arm of treatment. Since the PK profile of Avastin^®^ was linear for doses of 1–10 mg/kg (Avastin: EPAR-Scientific Discussion. EMA; Hettema et al., 2017; Wu et al., 2019), 1 mg/kg was selected to minimize drug exposure. The number of healthy male volunteers in this study was 50 per arm of treatment.

The mean concentration profiles of TAB008 Monoclonal Antibody Injection and Avastin^®^ were similar over the profiling interval. The treatment group ratios of LS geometric means for the three primary PK parameters were fully contained within the bioequivalence limits of 80–125%. These results were consistent with other similar studies in Caucasian, Japanese, Korean, and Chinese healthy volunteers (**Table 5**) (Knight et al., 2016; Hettema et al., 2017; Markus et al., 2017; Tajima et al., 2017; Hanes et al., 2018; Zhang et al., 2018; Cho et al., 2019; Wu et al., 2019; Zhang et al., 2019).

**Table 5:** Comparison of bevacizumab biosimilar and Avastin^®^ phase I clinical trials.

Trials	Subjects no.		Dose/IVI time		C_max_ (90% CI)	AUC_0-t_ (90% CI)	AUC_0-∞_ (90% CI)	TEAEs (%)		No. of ADA+ (IP/EU)
ABP215 (Markus et al., 2017)	IP*	EU	3 mg	90 min	103 (98–108)	96 (92–101)	96 (92–101)	IP*	EU	0/0
	68	67						27.0	15.0	
ABP215 (Hanes et al., 2018)	IP*	EU	3 mg	90 min	101.5 (94.6–108.8)	99.5 (94.1–105.3)	97.9 (91.4–104.9)	IP*	EU	-/-
	24	24						8.3	4.2	
PF-06439535 (Knight et al., 2016)	IP*	EU	5 mg	90 min	104.4 (98.4–110.8)	99.6 (93.7–105.9)	98.6 (92.2–105.4)	IP*	EU	2/1
	33	35						48.5	62.9	
BI-695502 (Hettema et al., 2017)	IP*	EU	1 mg	30 min	101.5 (92.7–111.1)	99.0 (91.0–108.0)	96.6 (885–105.3)	IP*	EU	-/-
	30	31						33.3	32.3	
BS503a (Tajima et al., 2017)	IP*	EU	3 mg	90 min	106.5 (100.9–112.5)	103.7 (98.2–109.6)	104.1 (98.0–110.5)	IP*	EU	0/0
	57	57						31.6	36.8	
BAT1706 (Wu et al., 2019)	IP*	EU	1 mg	90 min	99.1 (93.6–104.9)	104.8 (98.3–111.6)	105.1 (98.6–112.1)	IP*	EU	0/0
	42	43						55.0	48.8	
MIL60 (Zhang et al., 2018)	IP*	EU	3 mg	90 min	100.4 (97.0–111.0)	107.0 (101.0–112.0)	107.0 (101.0–113.0)	IP*	EU	1/1
	39	37						56.4	63.2	
IB1305 (Zhang et al., 2018; Zhang et al., 2019)	IP*	EU	3 mg	90 min	97.0 (90.0–104.0)	95.0 (89.0–101.0)	95.0 (89.0–101.0)	IP*	EU	2/2
	48	50						40.0	40.0	
CT-P16 (Cho et al., 2019)	IP*	EU	5 mg	90 min	103.0 (98.2–108.0)	104.3 (99.7–109.2)	103.9 (99.0–109.0)	IP*	EU	2/2
	45	47						19.6	42.6	

*IP is investigational product.

TEAEs considered related to the study drug in this study were reported for 19 (38.8%) subjects in the TAB008 group and 19 (38.0%) subjects in the Avastin^®^ group. AE seemed to vary widely between these studies (Knight et al., 2016; Hettema et al., 2017; Markus et al., 2017; Tajima et al., 2017; Hanes et al., 2018; Zhang et al., 2018; Cho et al., 2019; Wu et al., 2019; Zhang et al., 2019), but there was no significant correlation with the dose. The %AE with 1 mg/kg ranged across studies from 33.3% to 55.0% for biosimilars and 32.3% to 48.8% for Avastin^®^ (EU-sourced). The %AE with 3 mg/kg ranged across studies from 8.3% to 56.4% for biosimilars and 4.2% to 63.2% for Avastin^®^ (EU-sourced). The %AE with 5 mg/kg ranged across studies from 19.6% to 48.5% for biosimilars and 42.6% to 62.9% for Avastin^®^ (EU-sourced) (**Table 5**) (Knight et al., 2016; Hettema et al., 2017; Markus et al., 2017; Tajima et al., 2017; Hanes et al., 2018; Zhang et al., 2018; Cho et al., 2019; Wu et al., 2019; Zhang et al., 2019).

Here, we found that one subject in the TAB008 group and one in the Avastin^®^ group were positive for ADA after treatment; however, positivity disappeared upon subsequent testing. ADA levels were generally low or undetectable (previous studies report equally high values for both the biosimilar and originator).

## Conclusion

The present study shows that bevacizumab is pharmacokinetically similar to TAB008; indeed, the treatment group ratios of LS geometric means for the three primary PK parameters (C_max_, AUC_0-t_, and AUC_0-∞_) were fully contained within the bioequivalence limits of 80–125%. TAB008 Monoclonal Antibody Injection was safe and well tolerated by healthy Chinese male subjects (although evaluation of safety and tolerability at 1 mg/kg might not be sufficient to estimate the safety profile at the therapeutic dose), with no notable difference in immunogenicity.

## Data Availability

The raw data supporting the conclusions of this manuscript will be made available by the authors, without undue reservation, to any qualified researcher.

## Ethics Statement

The trial protocol was approved by the Drug Clinical Trial Ethics Committee of Beijing Shijitan Hospital of Capital Medical University.

## Author Contributions

JW and XW made substantial contributions to the conception and design of this study, to the acquisition of data, and to the analysis and interpretation of the data. LQ, LL, ZW, GC, YW, XL YL, HL, YT, CLi, and CLe made substantial contributions to the implementation of the study. All authors read and approved the final manuscript.

## Conflict of Interest Statement

The authors declare that the research was conducted in the absence of any commercial or financial relationships that could be construed as a potential conflict of interest.
